# Internal migrant preschoolers: life changes serve better emotion understanding?

**DOI:** 10.3389/fpsyg.2025.1628828

**Published:** 2025-11-20

**Authors:** Margarita Gavrilova, Aleksandra Karimova, Oksana Solopova, Aleksander Veraksa, Anastasia Yakushina

**Affiliations:** 1 Faculty of Psychology, Lomonosov Moscow State University, Moscow, Russia; 2 Faculty of History, Lomonosov Moscow State University, Moscow, Russia

**Keywords:** emotion understanding, TEC, internal migration, migrant children, preschoolers

## Abstract

This study investigates the impact of internal migration on emotion understanding in 6–7-year-old preschoolers, comparing children who relocated to Moscow within the past 3 years to their non-migrant peers. The study involved 182 neurotypical, native Russian-speaking children aged 6–7 from Moscow kindergartens, who were divided into a migrant (*n* = 71) and a non-migrant group (*n* = 111). Using the Russian adaptation of the Test of Emotion Comprehension (TEC), the research assessed three hierarchical levels of emotion understanding: external, mental, and reflective. Results showed that internal migrant children demonstrated significantly higher reflective emotion understanding, while no differences were found in external or mental levels. These findings suggest that migration-related challenges may foster the development of higher-order emotional competencies through exposure to diverse emotional experiences. The study interprets these results through Vygotsky’s concept of the social situation of development and Bronfenbrenner’s ecological theory. Despite its stressors, internal migration appears to serve as a developmental resource for enhancing emotion understanding in young children.

## 1 Introduction

Emotion understanding is the ability to understand the nature, causes, and consequences of one’s own and others’ emotions, and how to manage emotions in everyday life (Harris et al., 2016; Möller et al., 2022). Emotional understanding is one of the key components of emotional intelligence (the ability to perceive, express, understand, and manage emotions) (Salovey and Mayer, 1990). Emotion understanding in early years predicts future social and academic performance (Pauletto et al., 2021; Veraksa et al., 2020; Viana et al., 2020). As a child develops the ability to understand their own emotions, recognize the emotions of others, and regulate their emotional state, they become more confident and competent in social interactions, which enhances their school adjustment (Darling-Churchill and Lippman, 2016). Successful social adjustment, in turn, is a significant predictor of the ability to form close peer relationships (Jardine et al., 2022) and psychological well-being (Guerra-Bustamante et al., 2019; Pauletto et al., 2021; Ferragut and Fierro, 2012). For example, children who are good at emotion understanding are less likely to experience anxiety, depression and anger (Trentacosta and Fine, 2010). In adulthood, emotion understanding is required for a success in work and personal life (Brackett et al., 2004; Ruiz-Aranda et al., 2014).

Emotion understanding unfolds in stages, each with its own age boundaries. The stages of emotion understanding development are described in a well-known model by Pons and Harris (2005). This model delineates three hierarchical levels of emotion understanding. In the first stage (3–6 years), children learn to recognize emotions based on facial expressions and to understand the external causes of emotions. In the second stage (5–9 years), children realize that emotions can be caused not only by external, but also by internal psychological phenomena (for ex-ample, beliefs, memories, or expectations). In the third stage (8–11 years), children develop the understanding that two different emotions can be experienced simultaneously, and that morality affects one’s emotions. Additionally, in the third stage, they learn to regulate their emotions by means of cognitive strategies (Pons and Harris, 2005; Pons et al., 2004).

Emotion understanding is a complex, dynamic process that cannot be reduced to simple mechanical maturation of brain structures. From the perspective of L.S. Vygotsky’s approach, a key aspect of emotion understanding is the child’s active engagement in emotionally significant situations arising within their immediate social environment (Vygotsky, 1983). Shared emotional experiences create a social situation of development for the child (Veresov, 2024; Vygotsky, 1983).

Furthermore, many studies emphasize that the effective development of emotion understanding requires challenge, that is, a situation demanding that the child moves beyond their current capabilities (Blankson et al., 2012; Pons and Harris, 2019; Veraksa et al., 2020). This implies engaging the child in new social contexts and situations requiring the integration of multiple emotional perspectives simultaneously. Such challenges prompt the child to revise existing schemas and develop more flexible emotional representations (Pons and Harris, 2005; Veraksa et al., 2020).

Due to the fundamental role of emotion understanding, it seems important to study factors that influence the development of emotion understanding in preschool and elementary school children (Rocha et al., 2015). One such factor may be migration, which constitutes a significant challenge and novel social experience for both children and their families.

Rapid industrialization and economic growth in recent decades have led to increasing numbers of people migrating to big cities within their home countries to pursue higher education or better employment opportunities (Gordeeva et al., 2024; Lall et al., 2006). Globally, there are approximately 756 million internal migrants (i.e., those moving within national borders) (Lall et al., 2006; World Bank Group, 2016). Currently, the most pronounced rural-to-urban migration occurs in developing countries, particularly China, India, and Russia (Bhagat and Keshri, 2020; Charles-Edwards et al., 2019). For instance, in Russia, the total internal migration flow in 2022 reached 3,465.2 thousand people, including 1,644.2 thousand intra-regional migrants and 1,821 thousand inter-regional migrants. Of these, 69.1% originated from urban areas, while 30.9% came from rural localities (Russian Federal State Statistics Service, 2023).

When relocating to big cities, migrant children face social, economic, healthcare, and academic challenges in their new environment (Stevens and Vollebergh, 2008; Zhang et al., 2019). This relocation entails radical changes in their social situation, including shifts in peer networks, educational institutions, and living conditions. Such transformations inevitably affect children’s emotional well-being, as they involve the loss of familiar anchors, disruption of meaningful social ties, and the need to establish new relationships (Almazova et al., 2024; Andrade et al., 2023). Under these circumstances, migrant children experience heightened psychological stress, which may negatively impact their emotional adjustment, increase anxiety levels, and contribute to mental health risks (Sun et al., 2016).

The great amount of studies confirm that migrant children are at higher risk for emotional difficulties, including depressive symptoms, anxiety, and impaired social adaptation (Jaeger et al., 2012; Kouider et al., 2014; Zheng et al., 2022). For example, Stevens and Vollebergh (2008) found that migrant children from rural regions more frequently exhibit problem behaviors (e.g., aggression, hyperactivity) because of chronic relocation-related stress. Similarly, Kouider et al. (2014) demonstrated that children from migrant families often struggle with social integration, which correlates with increased loneliness and anxiety.

These findings underscore how migration poses unique developmental challenges for children’s emotional growth. From the perspective of Bronfenbrenner’s theory, migration profoundly restructures a child’s ecological system, necessitating the adoption of new social roles that accelerate emotional maturation and foster advanced empathy and intercultural skills. Within a supportive microenvironment, this transformation becomes a resource for developing a more complex and mature emotional competence than is achievable in a monocultural context (Bronfenbrenner, 1994).

However, as noted earlier, most studies focus on risks rather than potential adaptive resources, highlighting a critical gap in literature.

### 1.1 Current study

Thus, the present study aims to examine the characteristics of emotion understanding in 6-7-year-old preschoolers who have experienced relocation within their home country, compared to children without such experience. In order to achieve the aim of the study, we posed the following research question: Does internal migration affect the emotion understanding ability in 6-7-year-old preschoolers?

## 2 Materials and methods

### 2.1 Procedure

Prior to the study, ethical approval was obtained from the Ethics Committee of the Faculty of Psychology at Lomonosov Moscow State University (approval no: 2024/11). Written informed consent was acquired from all parents or legal guardians. Additionally, meetings were conducted with kindergarten administrators to explain the study’s organization and objectives. All participant data were anonymized using coded identifiers.

Concurrently with child assessments, caregivers participating children completed an online questionnaire (approximately 10 min duration). Caregivers answered the question about the family’s relocation experience over the past 3 years both within the region (from Moscow region to Moscow) and from another region. Following data collection, responses were analyzed to categorize children into two groups: (1) children whose families had relocated to Moscow from elsewhere in Russia within the past 3 years, and (2) children whose families had not relocated during this period.

### 2.2 Participants

The sample consisted of 182 typically developing children (93 boys, 89 girls) aged 6–7 years (M = 78.5 months; SD = 3.96 months) attending public kindergartens in Moscow. All participants were neurotypical and native Russian speakers. The sample was divided into: (1) migrant children (*n* = 71, 49% boys; families moved to Moscow within 3 years, 65% children relocated to Moscow from another region), and (2) non-migrant children (*n* = 111, 52% boys). Caregiver questionnaires indicated predominantly middle socioeconomic status: 90% of caregivers held university degrees, and 72% of families reported average income levels. Note that income data were based on caregiver self-reports rather than objective measures.

### 2.3 Measures

To assess children’s emotion understanding, the Russian adaptation of the Test of Emotion Understanding (TEC) (Pons and Harris, 2000; Veraksa et al., 2021) was used. The test materials consisted of illustrated storybooks, with separate versions for girls and boys. The experimenter read a story to the child, who then had to identify the appropriate emotion by pointing to one of four facial expressions. The TEC evaluates three hierarchical levels of emotion understanding: external level (The ability to recognize emotions, understand their external causes, and grasp how desires influence emotional state; e.g., *“Thes girl gets a birthday present. How does she feel at this moment?*”); mental level (the understanding of how beliefs and memories shape emotions, as well as the recognition of hidden feelings; e.g., *“Vanya has magnetic balls, but Kolya does not have such a toy. Kolya smiles because he does not want to show Vanya how he feels inside. How does Kolya really feel?”*); reflexive level (the understanding of mixed emotions, emotion regulation through cognitive strategies, and the impact of moral norms on emotional responses; e.g.*, “Maria looks at her new bike, which she has just been given. But she thinks she might hurt herself because she’s never ridden a two-wheeled bike before. How does Maria feel now?”*). Each level is scored from 0 to 3, yielding a total TEC score ranging from 0 to 9 (the sum of scores across all levels).

A questionnaire for caregivers was used to collect data about family migration to Moscow within the preceding 3-year period. The questionnaire also collected socio-demographic information (child’s sex and age, family income, and education) for sample description.

### 2.4 Data analysis

Analyses were conducted using Jamovi 2.3.28. The Shapiro–Wilk test (W) assessed normality assumptions. As data distributions violated normality, group comparisons employed the Mann–Whitney U- test; with effect sizes calculated using rank-biserial correlation (rb). Effect sizes were interpreted as: rb < 0.20 (negligible), 0.20–0.49 (small), 0.50–0.79 (medium), and ≥ 0.80 (large) per Cohen (2013) conventions. Measurement invariance was tested in JASP 0.95.2. Multigroup CFA (MGCFA) was applied to evaluate measurement invariance across groups. Configural invariance is considered confirmed with a Comparative Fit Index (CFI) ≥ 0.90; Standardized Root Mean Square Residual (SRMR) ≤ 0.08; and a Root Mean Square Error of Approximation (RMSEA) ≤ 0.06 When sample size is small (total N ≤ 300), the following cutoff criteria are suggested: for testing metric invariance, a change of ≤ − 0.005 in CFI, supplemented by a change of ≥ 0.010 in RMSEA or a change of ≥ 0.025 in SRMR would indicate noninvariance; for testing scalar invariance, a change of ≥ − 0.005 in CFI, supplemented by a change of ≥ 0.010 in RMSEA or a change of ≥ 0.005 in SRMR would indicate noninvariance (Chen, 2007).

## 3 Results

Table 1 presents descriptive statistics for emotional competence components and children’s age across two groups: (1) migrant children (children whose families relocated to Moscow within the past 3 years), and (2) non-migrant children (children without relocation experience).

**Table 1:** Descriptive statistics for TEC components and age in two groups of children.

Participants’ characteristics	Non-migrant children (*N* = 111)		Migrant children (*N* = 71)	
	M; SD	W; p	M; SD	W; p
Age, in months	78.8; 3.64	0. 981; 0.125	78.3; 4.69	0. 945; 0.004
TEC external	2.41; 0.623	0. 736; <0.001	2.58; 0.577	0. 677; <0.001
TEC mental	1.57; 0.696	0. 823; <0.001	1.65; 0.912	0. 876; <0.001
TEC reflective	1.23; 0.771	0. 845; <0.001	1.62; 0.763	0. 844; <0.001
TEC total	5.20; 1.306	0. 940; <0.001	5.85; 1.451	0. 956; 0.014

Results from measurement invariance testing indicated that configural invariance was supported (CFI = 1.00, RMSEA = 0.00, SRMR = 0.035), suggesting that the factor structure was consistent across groups. Metric invariance was achieved [CFI = 1.00 (ΔCFI = 0.00), RMSEA = 0.01 (ΔRMSEA = 0.01), SRMR = 0.034 (ΔSRMR = − 0.001)], demonstrating that factor loadings were equivalent across groups, confirming that the scale measured the construct similarly. Scalar invariance was also established [CFI = 1.00 (ΔCFI = 0.00), RMSEA = 0.01 (ΔRMSEA = 0.00), SRMR = 0.030 (ΔSRMR = −0.004)], allowing for meaningful latent mean comparisons across groups.

Table 2 presents the comparison between non-relocated and relocated (within 3 years) children in terms of emotion understanding. The results indicate that children who had relocated demonstrated significantly higher levels of emotion reflection ability and overall emotion understanding compared to non-relocated children, although the effect size was small (rb = 0,2,477). No statistically significant differences were found between groups in understanding external causes of emotions or mental state emotions.

**Table 2:** Differences in emotion understanding components in two groups of children.

Scale	Mann–Whitney U-test	*p*-value	Effect size
TEC external	U = 3,351	0.054	r_b_ = 0.1497
TEC mental	U = 3,755	0.565	r_b_ = 0.0471
TEC reflective	U = 2,964	0.002	r_b_ = 0.2478
TEC total	U = 2,965	0.004	r_b_ = 0.2477

## 4 Discussion

This study examined emotion understanding characteristics in 6-7-year-old preschoolers who experienced domestic relocation compared to their non-relocated peers.

Contrary to a substantial body of evidence linking migration experience to increased stress and risks to emotional well-being (Jaeger et al., 2012; Kouider et al., 2014; Sun et al., 2016), this study found that migrant children demonstrated significantly higher scale scores for reflective emotion understanding. Specifically, domestic relocated children showed enhanced emotion reflection abilities - they were better at identifying specific emotions triggered by events and distinguishing ambivalent emotional states. The discrepancies between our findings and those of previous studies, such as Bukhalenkova et al. (2022) and Voltmer and von Salisch (2019), may be attributed to the absence of language barriers in our sample, as all participants were native speakers. Specifically, while existing research associates migrant and bilingual status with greater difficulty in emotion recognition and understanding, our study focused on internal migrants without linguistic disadvantages, suggesting this factor may account for the divergent results.

This suggests that internal migration should be viewed not only as a vulnerability factor but also as a potential catalyst for the development of emotion understanding. These results align with findings indicating that low classroom quality in kindergarten groups may facilitate the reflective component of emotion understanding (Veraksa et al., 2020). It can be hypothesized that the experience of internal migration, similar to the experience of being in an overcrowded classroom with rigid rules, compels the child to actively utilize and consequently develop emotion understanding skills for successful adaptation. Comparable findings documenting higher levels of emotion understanding in children exposed to adverse factors have been reported in the scientific literature on parent–child relationships. For example, Yakupova and Suarez (2023) demonstrated that children of parents experiencing depression tend to develop enhanced emotion understanding capacity as they attempt to navigate the adult’s unstable condition. However, the authors note that despite the apparent positive nature of this finding, the high emotional load may become a foundation for depression in the long term (Yakupova and Suarez, 2023).

The absence of significant differences in external and mental levels of emotion understanding may indicate that the internal migration experience does not accelerate emotional development overall but exerts a selective influence on its most complex, culturally and socially mediated components. However, it is more probable that, according to the developmental periodization of emotions, the ability to understand internal and mental causes of emotions should, in most cases, already be formed at previous stages. In contrast, the development of the reflective component of emotion understanding, which is associated with comprehending mixed and moral emotions and emotion regulation, is actively developing at the age corresponding to the sample in this study.

The obtained results can be interpreted within Bronfenbrenner’s ecological model (Bronfenbrenner, 1994). Migration represents a radical restructuring of the entire ecological system. Intensive changes at the micro- and mesosystem levels, combined with the need to adapt to new cultural norms (macrosystem), create an environment for the migrant child that demands constant analysis and reflection on emotional rules and reactions. While the environment of the non-migrant child remains stable, the migrant’s experience involves continuous engagement in solving emotional and social tasks, which may accelerate the development of the currently maturing levels of emotion understanding. In addition to Bronfenbrenner’s ecological model, the results are explained through the concept of the social situation of development proposed within Vygotsky’s cultural-historical approach (Vygotsky, 1983). The act of moving entails changes in the child’s system of relationships with people, forms of activity and experiences, and alterations in their “worldview” (Vygotsky, 1983). The adaptation process of the entire family post-relocation likely leads to more frequent and open experiences and discussions of emotional states, causes of difficulties, and coping strategies, thereby stimulating the child’s comprehension of emotions, their origins, and regulation methods. In a broader context, this research contributes to the discussion of the “immigrant paradox” by specifically focusing on the aspect of emotional development, namely the ability to understand emotions (Coll et al., 2012). It demonstrates that the migration experience is non-linear and can simultaneously encompass both risk factors (stress, anxiety) and resources for development.

### 4.1 Limitations

The interpretation of the results must consider several limitations of this study. First, the correlational design does not allow for establishing causal relationships; pre-existing differences may exist between families who decided to relocate and those who did not. Second, we did not measure key parameters of the migration experience, such as stress levels, voluntariness of the move, or cultural distance, which could critically influence the outcomes. Third, we did not collect information about the reasons for the relocation, for example, escape poverty, natural disaster, act of terrorism, search for better opportunities or others. Fourth, the sample is focused on families in Moscow, which limits the generalizability of the findings to internal migrants in other regions.

## 5 Conclusion

The results indicate that the experience of internal migration can serve as a factor in the development of reflective emotion understanding in 6-7-year-old children. Thus, internal migration represents a complex phenomenon combining both stress factors and developmental potential. In explaining the higher scale scores for reflective emotion understanding among migrant children, we rely on the notion that the necessity to cope with complex social situations drives the development of emotion understanding (Veraksa et al., 2020); however, this outcome should not be considered unequivocally positive, as it is likely short-term. Longitudinal studies are required to track whether this short-term advantage is offset by long-term negative consequences. Such consequences may include chronic stress, emotional burnout, increased anxiety, and difficulties in establishing trusting relationships in the long-term perspective.

The results of our research propose that educators and caregivers can view internal migration not only as a risk factor, but also as a context for focused interventions that tap into children’s adaptive potential. Future research should explore how kindergartens and schools can utilize these developmental opportunities to enhance the resilience of migrant children. The obtained results can also be applied in the development of educational programs for teachers in kindergartens and psychological interventions for relocated families.

## Data Availability

The raw data supporting the conclusions of this article will be made available by the authors, without undue reservation.
